# Radiometric Calibration and Uncertainty Analysis of KOMPSAT-3A Using the Reflectance-Based Method

**DOI:** 10.3390/s20092564

**Published:** 2020-04-30

**Authors:** Cheonggil Jin, Hoyong Ahn, Doochun Seo, Chuluong Choi

**Affiliations:** 1 Division of Earth Environmental System Science (Major of Spatial Information Engineering), Pukyong National University, Busan 49513, Korea; cgjin@pukyong.ac.kr (C.J.); hyahn85@korea.kr (H.A.); 2 Department of Agricultural Biology, Climate Change and Agro-Ecology Division, National Institute of Agricultural Sciences, Rural Development Administration, Jeonju 55365, Korea; 3 Image Data System Development Division, National Satellite Operation & Application Center, Korea Aerospace Research Institute, Daejeon 34133, Korea; dcivil@kari.re.kr

**Keywords:** absolute radiometric calibration, reflectance-based method, radiative transfer code (RTC), KOMPSAT-3A, calibration coefficient, uncertainty, calibration and validation (Cal/Val), digital number (DN)

## Abstract

In recent years, Korea has sustained consistent access to remote sensed data by launching Korea Multi-Purpose Satellite-3A (KOMPSAT-3A, K3A)—an updated version of the high-resolution KOMPSAT series. This KOMPSAT-3A required calibration and validation (Cal/Val) before and after its launch to enable proper functional characterization and to maintain the veracity of data collected. The Korea Aerospace Research Institute (KARI) executed the initial prelaunch calibration in the laboratory and we performed the Cal/Val of KOMPSAT-3A during the Launch and Early Operation Phase (LEOP) in the field. Two suitable sites in Korea and Mongolia with stable weather, almost uniform terrain, and near Lambertian diffusion, provided the necessary tarp reflectance to calculate the absolute radiometric calibration coefficients. The surface reflectance was determined using 12 and four well-calibrated reference reflectance tarps employing the FieldSpec^®^ 3(Analytical Spectral Devices Inc., Boulder, CO, USA) Spectroradiometer. Subsequently, the top of atmosphere (TOA) radiance was estimated using radiative transfer code (RTC) software based on the Atmospheric and Topographic Correction (ATCOR). In addition, cross calibration was simultaneously performed at the Libya-4 pseudo invariant calibration site (PICS) for KOMPSAT-3A TOA radiance, using the spectral band adjustment factor (SBAF) compensated Landsat 8 reflectance and the Second Simulation of Satellite Signal in the Solar Spectrum (6S) to compute cross calibration coefficients. The results of the KOMPSAT-3A absolute calibration coefficient show that the R^2^ values were over 0.99, implying a significant correlation for almost all bands between the TOA radiance and the KOMPSAT-3A spectral band response at both campaign sites. However, this study reveals a difference of less than 5% calibration gains for all bands compared to the prelaunch values, while the cross calibration gain is below 5% in visible bands and above 5% in the near infrared band. An effort to optimize the reliability of the absolute calibration coefficients resorted to the rigorous quantification of uncertainties amongst atmospheric conditions, the digital number (DN), the reflectance tarp, the bidirectional reflectance distribution function (BRDF), and ozone levels. Therefore, we presumed that the total uncertainty was 4.27%, which conforms to some published results.

## 1. Introduction

Korea Multi-Purpose Satellite (KOMPSAT) is the name of the satellite series that was developed to satisfy the rapidly increasing demand for high-resolution satellite images due to economic development. The Korean government successfully launched a new satellite, named Korea Multi-Purpose Satellite-3A (KOMPSAT-3A), on 26 March 2015. The Korea Aerospace Research Institute (KARI) performed calibration and validation (Cal/Val) to enable an appropriate radiometric quality in the Launch and Early Operation Phase (LEOP). This Cal/Val comprised geometry (position), spatial (resolution), and radiometric (energy) elements.

Absolute radiometric calibration aids in investigations of the liaison between radiance, reflectance, and the digital number (DN), with the aim of extracting the reflectance of targeted scenes on the surface [1]. During the absolute calibration coefficient process, the DN is converted into radiance. We performed this exercise using onboard calibration sources, which are considered to be well-established [2]. However, regardless of the satellite prelaunch calibration data quality, the sensor is sensitive to post-launch changes; even when the sensor is stable or properly characterized, evaluations must be continued during the post-launch period. Therefore, post-launch calibration using onboard calibration systems is necessary, and sensors are continually monitored to identify issues and maintain data quality. In other words, the image quality is managed by absolute calibration using a vicarious approach with calibration devices installed in the sensor or by measuring the site surface reflectance [3]. Absolute calibration using a vicarious approach is conducted under the assumption that the DN of the satellite sensor is linear to the top of atmosphere (TOA) radiance.

Three methods have been developed to vicariously calibrate multispectral optical sensors in orbit [4,5], and have been successfully used for the Landsat Series [5,6], IKONOS, and SPOT HRV [7]. The reflectance-based calibration method has mostly been used in the context of satellite sensors as a method of monitoring radiometric calibrations [3,5,6,8,9]. Reflectance-based methods involve imaging a relatively homogeneous Region of Interest (ROI) in tandem with ground-based measurements [9].

After launching the KOMPSAT series, radiometric calibration and validation was applied to the imagery by the Korean Aerospace Research Institute (KARI) and Pukyong National University (PKNU) [10,11]. This paper examines the laboratory-based prelaunch calibration conducted by KARI, together with the post-launch absolute calibration and cross calibration executed by a tag team of KARI and PKNU scientists during the LEOP. The research was performed in three main phases:

1. Using a well-calibrated reference reflectance tarp, we conducted absolute radiometric calibration in Mongolia (Zuunmod) and South Korea (Goheung). Employing each site’s surface reflectance data, we calculated the TOA radiance by the ATCOR [12] radiative transfer code (RTC). Then, we analyzed and computed the absolute coefficients for all of the KOMPSAT-3A bands;
2. We converted the calibrated DNs to TOA radiance using KOMPSAT-3A absolute coefficients. Additionally, we compared these values with the Second Simulation of Satellite Signal in the Solar Spectrum (6S) TOA radiance based on the spectral band adjusted Landsat 8 reflectance for KOMPSAT-3A at the Libya-4 Pseudo Invariant Calibration Site (PICS). Then, we divided 6S radiance by DN and extracted the calibration coefficients for radiometric validation;
3. We estimated the uncertainty of absolute radiometric calibration geared towards understanding the connection between individual factors and the overall uncertainty.

## 2. Methods 

### 2.1. Study Flowchart and Image Selection

In this study, we employed a reflectance-based method for KOMPSAT-3A absolute radiometric calibration. This approach has previously been used for the optical satellite’s radiometric function [13]. We conducted a six-phase field campaign in Goheung, South Korea (May, 2015), and in Zuunmod, Mongolia (June, 2015), whereby the tarp reflectance needed to calculate the absolute calibration coefficient was measured. The calculations based on fieldwork reflectance and KOMPSAT-3A images are presented in Table 1.

**Figure 1:**
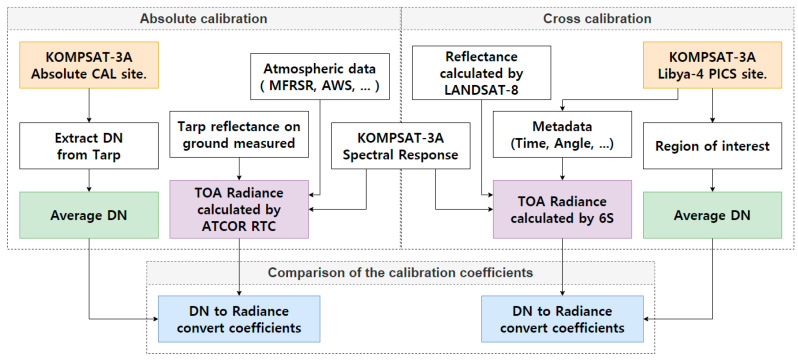
A flow chart of the three main steps of this study.

We installed a Multi Filter Rotating Shadow band Radiometer (MFRSR), PTU-2000 [14], and Automatic Weather System (AWS). This MFRSR was used to quantify direct, diffuse, and total solar irradiance and the PTU-2000, together with AWS, recorded the weather parameters (i.e., temperature, air pressure, visibility, wind direction, speed, and humidity). The reference tarp reflectance obtained from fieldwork and image-based DN were used to estimate the TOA radiance via RTC considering MFRSR, AWS, and PTU-2000 uncertainty corrections. The absolute calibration coefficients were calculated using linear regression. Equally, cross calibration was carried out for KOMPSAT-3A using SBAF corrected Landsat 8 reflectance and 6S simulation at the Libya-4 PICS site to determine the cross calibration coefficients. In addition, to ensure the reliability of the absolute calibration coefficients, uncertainty was estimated based on topographic effects and atmospheric conditions considering reflectance tarp measurement deviations, DN homogeneity, the bidirectional reflectance distribution function (BRDF), and ozone levels (Figure 1). 

### 2.2. KOMPSAT-3A Sensor Overview

After launching KOMPSAT-3 in 2012, KOMPSAT-3A was successfully deployed on a Dnepr Rocket at the Yasny launch base in Orenburg Oblast, Russia, on 26 March 2015. KOMPSAT-3A is equipped with a multispectral imaging ground sample distance (GSD) of 2.2 m and an AEISS-A sensor with a 0.55 m panchromatic GSD and 5.5 m thermal infrared imaging GSD. KOMPSAT-3A’s data quantization is 14 bit and shares the same platform and payload as KOMPSAT-3. The same optical multispectral sensor is mounted in KOMPSAT-3 and KOMPSAT-3A as an AEISS and AEISS-A sensor. KOMPSAT-3A has an orbit inclination of 97.51 deg and an orbital period of 98.5 min. It weighs 1100 kg, and is 2.0 × 3.8 m in the launching case and 2.0 × 3.8 × 6.3 m in orbit. In addition, it is operated with a sun synchronous orbit elevation of 528 km and a ground swath width of 12 km. 

We received KOMPSAT-3A images (52 scenes) from 1 Feb 2016 to 27 Oct 2019. However, the upper tilt ±15 deg images displayed many uncertainties; thus, high tilt images (28 scenes) were discarded and only lower tilt ±15 deg images (24 scenes) were used. The selected images’ pass time was 11:36:33 ± 0:05:11 (UTC) and tilt angle was 3.60 ± 9.64 deg.

**Figure 2:**
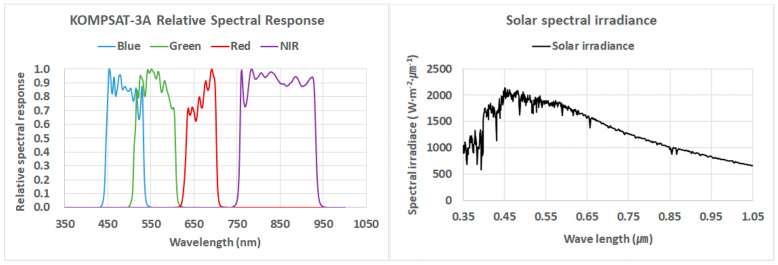
The relative spectral response (RSR) of Korea Multi-Purpose Satellite-3A (KOMPSAT-3A) and the solar spectral irradiance.

Multispectral remote sensors are used to record the reflected, radiated, and scattered energy from multiple bands of the electromagnetic spectrum from the reference target. One often-overlooked factor is the effect of the relative spectral response (RSR) of a sensor on broadband spectral measurements. The RSR explains the quantum efficiency of a sensor at a specific wavelength of each band. The exoatmospheric radiance changes due to the effects of RSR, exoatmospheric solar illumination, surface reflectance, and atmospheric transmittance [15]. The KOMPSAT-3A radiometric characteristics illustrated in Table 2 are based on the RSR function (Figure 2). The nominal bandpasses were based on instrument design wavelengths, while the 50% bandpasses were determined from actual KOMPSAT-3A sensor response curves. 

6S is a computer simulation program for the basic and advanced RTC used with KOMPSAT-3A. This program simulates the reflection of the solar spectrum considering spectral, geometrical, and atmospheric conditions. Therefore, 6S atmospheric correction for KOMPSAT-3A was able to remove the effects of the atmosphere. In this case, the Landsat 8 reflectance with SBAF compensation for KOMPSAT-3A was used. 

According to other studies, the visibility, water vapor, and aerosol optical depth (AOD) in Libya-4 PICS were 37~40 km [16], 0.11 ± 0.06, and 2.8 ± 0.7 g·cm^−2^ [17]. Therefore, the radiance and reflectance at Libya-4 PICS (28.9047N, 23.7889E) were simulated 34,587 times for 183 days, a 2-day interval (KOMPSAT-3A average pass time), 7 tilt angles (−18, −12, −6, 0, 6, 12, and 18 deg), 3 water vapors (2.1, 2.8, and 3.5 g·cm^−2^), 3 ozone values (0.28, 0.31, and 0.34 g·m^−3^), and 3 visibilities (20, 30, and 40 km) for each season.

### 2.3. Field Campaign for Radiometric Calibration

Field campaign test sites for absolute calibration using a vicarious approach require specific conditions. First, the terrain must be uniform, without any unusual topography. Second, the backscatter factor of the terrain must be small and consistent, as backscatter factors with high differences make precise reflectance tarp measurements difficult. Third, the climate should be considered; for example, steppes in Mongolia are arid areas adjacent to desert, with many areas surrounded by desert. In addition, the area must have a low surface roughness (a meter-scale longitudinal roughness of up to 10–20 cm), a lower gradient (≤20%), no snow, a slow wind velocity (≤3.4 m/s), and a high altitude (1000 m above sea level), and be far from the shore lines (≥1000 km) [3,18,19]. 

The field campaign was conducted in Mongolia and Korea during the LEOP of KOMPSAT-3A with specially crafted reflectance tarps. The Zuunmod site is 24km south of Ulaanbaatar in Mongolia, with suitable weather and topographic conditions, and has a good transportation network. Alongside the Mongolian local team, we installed six ground targets (3.5%, 23%, 32%, and 53% reflectance tarp with black and white edges). 

A potential fieldwork site in Korea with adequate weather and topographic conditions was seemingly difficult to find. However, after having conducted a serious search, we identified a feasible field campaign site for absolute calibration at the KARI Goheung Aviation Center. This site is neither the best nor worst, but has a big airplane hangar and warehouse, which permitted us to install twice as many targets (3.5%, 5%, 15%, 23%, 25%, 30%, 32%, 40%, 45%, 53%, 60%, and 70% reflectance tarp) as at the Mongolia site.

**Figure 3:**
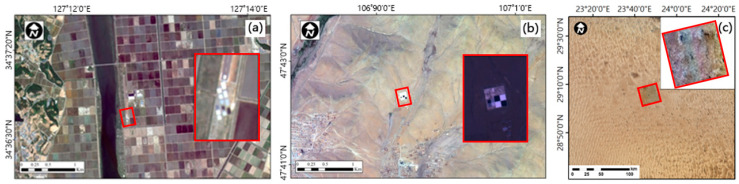
The field campaign site. (**a**) Goheung site on 27 May 2015; (**b**) Zuunmod site on 17 June 2015; and (**c**) Libya-4 pseudo invariant calibration site (PICS) used for the cross calibration.

From 26 May 2015 to 28 May 2015, twelve reflectance reference tarps were installed at the Goheung site, while four reflectance reference tarps were installed at the Zuunmod from 17 June 2015 to 19 June 2015. The surface reflectance was measured with a Fieldspec 3 Spectroradiometer over 3 days (Figure 3a). A total of four observations were conducted before and after the satellite overpass (two times each) and the spectral reflectance was calculated as an average of these measurements. 

The surface reflectance was equally measured on the edge of a target colored white and black to account for the insufficient number of reference tarps. In total, four observations were conducted before and after the satellite overpass times. In order to compute for BRDF compensation, we measured the same satellite tilt angle reflectance. Additionally, the weather parameters were measured with a portable weather system supplemented with information from the Ulaanbaatar weather station. 

### 2.4. Converting Surface Reflectance to TOA Radiance

TOA radiance can be calculated from the surface reflectance with RTC in ATCOR, which is in turn used to measure the indirect and direct path radiance at the satellite overpass time and to remove any geometric and atmospheric impact on ROI [20]. We measured the fieldwork site’s reference tarp reflectance, radiometric, and atmospheric data, and inputted all the data into the RTC to calculate the predicted radiance [21,22]. The atmospheric data (Table 3) were collected during the fieldwork using MFRSR and AWS, but the aerosol data were extracted via the standard atmospheric model in ATCOR.

In addition, the spatiotemporal properties of the area, sensor spectrum profile, and atmosphere attribute profile are required for absolute calibration in the vicarious approach [23]. Water vapor was estimated based on other data measured during the field campaign, including the atmospheric pressure, air temperature, and humidity. Ozone was estimated from measurements collected on the same day using an Ozone Monitoring Instrument (OMI). Table 3 enumerates the atmospheric parameters inputted into the ATCOR. 

### 2.5. Cross-Validation at Libya PICS-4

The best sites for radiometric cross calibration are pseudo invariant sites with a homogenous radiometric, slight weather change, a flat surface, and a high reflectance. A stable desert is suitable for a pseudo invariant site [3] and PICS sites that have been recommended are Libya, Algeria, Niger, and Mauritania [24]. Additionally, surface topographic effects not corrected directly and indirectly affect the reflectance by the atmospheric condition [25].

Therefore, the radiometric cross calibration coefficients were calculated using the TOA radiance from KOMPSAT-3A and 6S bearing the same RSR. Then, these values were used to estimate TOA reflectance with geometrically correcting KOMPSAT-3A at a well-known Libya-4 site.

The reason why reflectance is used is because the TOA radiance displays a lot of solar irradiance caused by the overpass date and time. However, the use of normalized solar radiance and irradiance can enable the abatement of solar illumination and atmospheric interference via the sensor’s TOA reflectance.

When we compared the data with the other sensor’s radiometric quality, the results demonstrated that the TOA reflectance subdued the effect of solar zenith angles stemming from time differences. We can change the KOMPSAT-3A TOA reflectance to radiance by Equation (1):

(1) $$\rho_{\lambda }=\frac{\pi \cdot L_{\lambda }\cdot d^{2}}{{ESUN}_{\lambda }\cdot cos\theta_{s}}$$

where $\rho_{\lambda }$ is the TOA reflectance, $L_{\lambda }$ is the KOMPSAT-3A TOA spectral radiance (W∙m^−2^∙sr^−1^), ${ESUN}_{\lambda }$ is the average solar exoatmospheric spectral irradiance (W∙m^−2^∙band^−1^), $\theta_{s}$ is the sun zenith angle (deg), and d is the ratio of Earth Sun distance.

The 6S atmosphere parameters (visibility, AOD, and water vapor) at the Libya-4 PICS were 37~40 km [16], 0.11 ± 0.06, and 2.8 ± 0.7 g·cm^−^^2^ [17].

## 3. Results and Discussion

### 3.1. Radiometric Absolute Calibration

In this exercise, we used both the reflectance values that came from the Mongolian and Korean fieldwork data and DN (Min, Max, Mean, and Stdev) resulting from a zonal analysis of KOMPSAT-3A L1R imagery in an ROI polygon. The TOA radiance has a first order relation with DN, and can thus be calculated via the regression analysis of DN and Radiance Equation (2):

(2) $$L_{\lambda }=Gain\times DN,$$

where $L_{\lambda }$ is the band’s spectral radiance obtained using ATCOR and *Gain* is the absolute calibration coefficient for each band [26].

Therefore, the uncertainty of *Gain* is the radiance sensitivity from the image DN [27].

The KOMPSAT-3A image data were converted from DN to TOA radiance by Equation (3):

(3) $$p_{\lambda }^{\prime }=\mathrm{DN}\cdot \mathrm{Gain}+\mathrm{Offset},$$

where $p_{\lambda }^{\prime }$ is the TOA spectral reflectance, DN is the pixel value, Gain is the scaling factor for the band, Offset is the bias factor, and $\theta_{s}$ is the Solar Elevation Angle (deg).

It should be noted that the TOA reflectance is used to normalize solar irradiance, minimize illumination differences, and correct atmospheric and topographic effects.

It was assumed that the changes between TOA radiance and sensor characteristics were linear. Each observation was performed to estimate the regression parameters using least squares fitting. 

As in Equation (3), the linear regression has Gain and Offset, and one can use "only Gain” and/or “Gain & Offset”. However, in order to eliminate Offset values, KARI recurrently measured dark counts [9]. Therefore, the result of the Offset value was very small and we set the condition to “0” Offset. 

**Figure 4:**
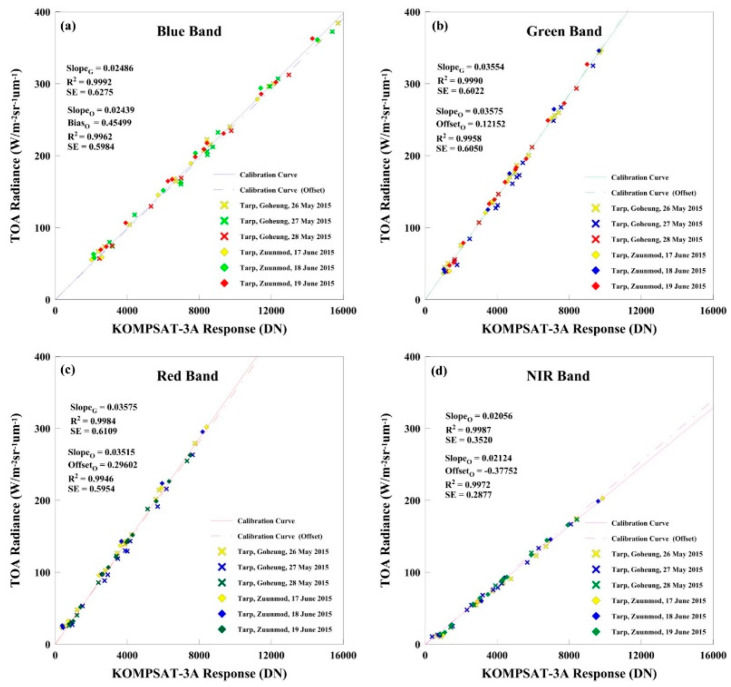
Absolute calibration conducted using a vicarious approach.

Two calibration coefficients (zero Offset and Gain & Offset) were estimated and the results were compared. The standard errors of the regression for both methods were significant (α = 0.05) based on the *t*-test. Figure 4 shows the final calculated coefficients obtained for both methods.

The results of absolute calibration coefficients for KOMPSAT-3A are shown in Table 4. The linear regression analysis significance level was lower than 5%. The correlation coefficient for each day (6 days) and band (4 bands) was greater than 0.99. 

Daily Gain was compared for 26 May 2015 at Goheung and 17 Jun 2015 at Zuunmod; the difference in the bands’ absolute calibration coefficients was small for each field campaign site. Both cases achieved a significant level based on the t-test. This is likely due to the incidence angle and atmospheric uncertainty during satellite overpass.

### 3.2. Calibration Coefficient Assessment

The difference of absolute calibration coefficients for the field campaign sites was lower than 1.5% (Blue, Red, and NIR) and 3% (Green). The calibration uncertainty was 2.5% in the visible and NIR band. All Gain values were in agreement within ±3%, indicating a good consistency, as shown in Table 5. 

The estimated KOMPSAT-3A absolute radiometric calibration coefficient and correlation coefficient (R^2^) values were 0.02486 and 0.9992 (Blue), 0.03554 and 0.9990 (Green), 0.03575 and 0.9984 (Red), and 0.02056 and 0.9986 (NIR), respectively. For all bands, the R^2^ values of the linear regression were over 0.99. The Gain coefficients were < 0.05, as obtained by the t-test, and the correlation between TOA radiance and DN in Mongolia (Zuunmod) and Korea (Goheung) for the KOMPSAT-3A reached a significant level. 

Table 5 shows the absolute Gain coefficients estimated in this study, from the KOMPSAT-3A prelaunch and from a cross-calibration [10], as well as the ratio of the absolute coefficients.

### 3.3. Radiometric Cross-Validation Using Libya-4 PICS

**Figure 5:**
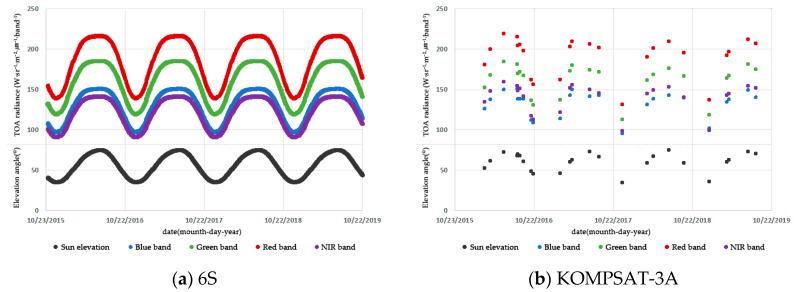
The TOA radiance trend in Libya-4 PICS obtained by 6S (**a**) and KOMPSAT-3A (**b**).

Figure 5 and Table 6 demonstrate a radiance similarity of 91.91%~104.89% between KOMPSAT-3A and 6S, with a correlation of 0.9168~.9572 in all bands. The KOMPSAT-3A radiance difference was −4.15~11.77 (W·sr^−1^·m^−2^) and −3.1%~8.4%. The absolute calibration radiance in visible bands was −3.1%~4.7% lower than that of 6S, while the NIR was 8.4% greater than that of 6S. The visible bands’ result can be associated with the satellite pass time and tilt angle effects.

**Figure 6:**
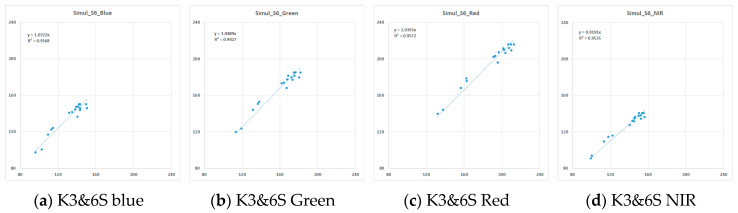
Measured by KOMPSAT-3A (K3A) and simulated by 6S over the Libya-4 PICS TOA radiance (X: front, Y: rear).

According to Figure 6, there was some degree of similarity in the KOMPSAT-3A radiance in every band. The resultant calibration coefficients for KOMPSAT-3A and 6S are presented in Table 6. The similarity for visible bands satisfied the significance level of 5%, whereas that of the NIR band did not attain the significance level of 5%.

We compared the TOA reflectance of Landsat-8 and KOMPSAT-3A at the Libya-4 PICS (Table 7). Without spectral band adjustment factor (SBAF) compensation, the blue, green, red, and NIR band TOA reflectance differences of KOMPSAT-3A and Landsat-8 were within −1.19%, −3.65%, −7.78%, and −22.04%, respectively, at the Libya -4 PICS. This reflectance difference came from the band RSR of the two sensors. In particular, the Landsat-8 and KOMPSAT-3A sensor NIR RSR significantly differed. After SBAF compensation, the reflectance difference was lower than 5%, and the SBAF coefficients between Landsat-8 and KOMPSAT-3A were 0.976, 1.014, 1.023, and 1.221, respectively. This explains the lower TOA reflectance of KOMPSAT-3A in the NIR band than that of Landsat-8 [28]. 

The TOA radiance at the Libya-4 PICS was calculated using the result of the calibration coefficients of KOMPSAT-3A, determined via 6S simulation and SBAF compensated Landsat 8 reflectance. The percentage differences are shown in Table 8. 

The absolute calibration image scanning mode was “1” during LEOP and the cross calibration mode was “2” at the Libya-4 PICS. The value of the blue band is the same in both modes, while the other bands’ coefficients are doubled in mode 1. The difference in the NIR band was 8.4% at the Libya-4 PICS. This can be explained by the Time Delay Integration (TDI) setting values in the RSR of sensors. If the TDI mode changes, the sensor’s signal noise ratio (SNR) and modulation transfer function (MTF) value change [29]. Moreover, the KOMPSAT-3A has a 940 nm NIR band, making it vulnerable to water vapor absorption. 

### 3.4. Comparison of the Calibration Coefficients 

As illustrated in Table 9, the calibration Gains obtained in this study with the prelaunch Gains revealed <5% difference for all bands. Cross calibration Gain was <5% in the visible band and >5% in the near infrared band.

Comparatively, the calibration results of IKONOS [30] underwent an on-orbit acceptance test under conditions of ~10% of a reflective target with a 30 deg Sun elevation angle. Therefore, the <5% difference in this study demonstrated excellent agreement. In general, the cross-calibration differences were 8% and 11% in the Red and NIR bands, respectively; noting that their bandwidths display very different RSR in sensors. The KOMPSAT-3A radiometric Cal/Val results [31] agreed with those of the aforementioned study, with differences in TOA reflectance based on an RSR of 6% and 8% for the Red and NIR bands respectively. 

The variance in the simulated 6S coefficients resulted from the difference in chapter 4.4 (BRDF). In order to minimize BRDF, path, and diffuse radiance effects, we used only cloud-free KOMPSAT-3A images with a tilt angle of less than 15 deg. The aerosol and water vapor effect was accounted for a represented in chapter 4.3.

## 4. Uncertainty Analysis

Uncertainty is an important index that represents the accuracy of results; thus, as the uncertainty value becomes lower, the results approach the ground truth. In general, the uncertainty of the reflectance-based vicarious calibration method is determined by assessing the accuracy of each factor, to estimate the overall uncertainty [1]. In this study, five factors constituted the uncertainty in the field campaign: tarp reflectance measurement difference, DN homogeneity, BRDF, atmospheric conditions (visibility and water vapor), and ozone. Assuming that these factors were mutually independent, we calculated the overall uncertainty using the Root Sum Squares (RSS) analytical method [32].

### 4.1. Tarp Reflectance Measurement

The Fieldspec accuracy depends on the calibration accuracy. For the DN ratio, device error is eliminated through dark current adjustment, except for irregular errors that are difficult to measure. In this study, the accuracy of the observed tarp reflectance (each observation, 10 measurements) was calculated after the solar zenith angle correction and standard deviation were accounted for. From the results, the uncertainty was ~1.9% and appeared to be increasing inversely to the reflectance. Each daily tarp uncertainty manifested similar significant differences. However, the reflectance tarps 5%, 15%, and 53% used at Goheung exhibited relatively high deviations, suggesting that they should be replaced so as to obtain precise measurements. On May 26 and June 19, the measured tilt angle was equal to the large zenith angle. However, our spectrometer target pistol measured nadir and the same satellite tilt angle for BRDF correction [33].

### 4.2. The Uncertainty of DN from the Tarp

To assess the homogeneity of each reference tarp, the uncertainty of the images was calculated from the DN. In this calculation, the standard deviation of the DN was calculated for the tarp center according to the reflectance of a 2 × 2 pixel (four pixels in total). 

It should be noted that brand new tarps have uniform reflectance rates, while used tarps have dirt and stains arising from storage. Additionally, tarp reflectance uniformity differs because of bottom conditions (ex, grass, asphalt, and sand, etc.). Therefore, it is advisable to check the DN and STdev for obtaining the tarp reflectance quality based on the satellite image before using the reflectance tarp. Korea site bottom conditions comprised a mixture of grass, concrete, asphalt, and gravel, whereas the Mongolia site had only concrete. However, the DN uncertainty was 2% and the Goheung site had a relatively higher deviation than the Zuunmod site. This result was likely influenced by the number of tarps measured, tarp degradation, and the tarp size. The deviation of the DN for each band is demonstrated in Table 10. DN uncertainty was similar among all tarps. However, the 5%, 30%, and 60% reflectance tarps displayed relatively higher deviations, likely affected by the mutual interference of pixels due to the small tarp size (10 × 10 m). 

In the ATCOR BRDF Compensation module, we calculated no BRDF and empirical BRDF correction mode(II). The uncertainty of BRDF comes from the difference between no BRDF and Empirical BRDF correction [12].

### 4.3. The TOA Radiance Uncertainty Based on the Atmospheric Simulations 

In this study, the AWS, MFRSR, and UV-MFR were used to calculate the water vapor (H_2_O), ozone column, and aerosol optical depth (AOD) at the Goheung site. For daily uncertainty, 28 May 2015 had the largest deviation due to unstable atmospheric conditions caused by rainfall that morning. At the Zuunmod site, UV-MFR was not installed, coupled with an insufficient time to generate data for Langley plots, rendering the conventional ozone and aerosol measurements almost impracticable. However, the OMI method emerged as an appropriate alternative ozone measurement. Table 3 states the atmospheric parameters used in the ATCOR. The results in Table 11 represent the TOA radiance uncertainty based on the atmospheric conditions prevailing during the field campaign (Table 3).

Generally, the uncertainty of the RTC model was 1.4%~7.2% [34] but during this exercise, field-measured atmospheric data and the nearest AWS data were exploited, yielding a difference in the atmospheric condition’s results. Therefore, we simulated each the TOA radiance and calculated uncertainty using ATCOR from the field atmospheric measurements and the nearest AWS data.

The uncertainty related to ground reflectance increased as the reflection increased. Band deviation was ordered from short wavelength as Blue, Green, Red, and NIR. The overall uncertainty dependent on atmospheric conditions was ~0.614%. This can be improved by using rigorous uncertainty calibrations when calculating the atmosphere transmittance, aerosol optical thickness, and ozone with MFRSRs, UV-MRFs, sun photometers, and sky radiometers. 

### 4.4. BRDF

The BRDF was an important parameter in the RTC for the reflectance-based vicarious calibrations that affected the calibration accuracy. It should be noted that most botanical surfaces have non-Lambertian reflection characteristics [35]. Additionally, KOMPSAT-3A had to be corrected for the BRDF effect relative to the position of the sun and satellite. The calculated BRDF uncertainty was within 0.6%, which increased as the tarp reflectance increased. The uncertainty for each band increased in the order of NIR, Red, Blue, and Green (Table 10). 

On 26 May 2015 and 17 June, 2015, the satellite tilt angle exceeded 15 deg and BRDF correction [36] was applied when converting tarp reflectance to TOA radiance. This caused a high daily BRDF uncertainty (1.23%) on 29 May 2015. 

### 4.5. Total Uncertainty 

Based on the uncertainty calculated for each factor, the total uncertainty was calculated using Equation (4),

(4) $$\sigma_{0}=\sqrt{\sigma_{Ref}^{2}+\sigma_{DN}^{2}+\sigma_{ATM}^{2}+\sigma_{BRDF}^{2}+\sigma_{Ozone}^{2}},$$

where $\sigma_{Ref}^{2}$ is the tarp reflectance measurement difference, $\sigma_{DN}^{2}$ is satellite DN homogeneity, $\sigma_{ATM}^{2}$ is atmospheric condition, $\sigma_{BRDF}^{2}$ is the bidirectional reflectance distribution function, and $\sigma_{Ozone}^{2}$ is the ozone content.

This indicates the standard deviation of the true value from the calculated value and results are Table 12. The ozone content was used after measuring with the OMI. In general, the OMI ozone uncertainty was within 3% [37].

The total uncertainty with respect to the campaign site was 5.39% and 3.73% for Goheung (28 May 2015) and Zuunmod (17 June 2015), respectively. The overall total uncertainty was 4.27% and increased for each band as follows: Blue, Green, NIR, and Red.

The total uncertainty results from this study are in agreement with the literature. In two studies, the reflectance-based vicarious calibration of uncertainty was estimated to be ~5% [38]. Similarly, the uncertainty of cross-calibration was ≤5% between a well-calibrated sensor and a reference sensor using PICS [39]. 

For the uncertainty results of the reflectance tarps (3.5%, 23%, 32%, and 53%) at the Goheung and Zuunmod sites, significant uncertainty was similar, except for the 3.5% reflectance tarp, which showed an inverse relation between uncertainty and reflectance. This was similar to the in-flight calibration results obtained using the Monte Carlo method [1].

Since the ozone uncertainty in this study was set to 3% [37], it is imperative that rigorous ozone uncertainty calculations be subsequently conducted; thus, for future radiometric calibrations, AOD and ozone levels measured with a sky radiometer and sun photometer are needed to better analyze atmospheric conditions. 

## 5. Conclusions

This study establishes the relationship between the DN and radiance exploiting radiometric calibration coefficients obtained via KOMPSAT-3A absolute radiometric calibration.

We applied reflectance-based absolute radiometric calibration for KOMPSAT-3A, whereby six field campaigns were conducted in South Korea (May 2015) and Mongolia (June 2015) to compute absolute calibration coefficients via reflectance tarps. This vicarious approach displayed a significance level below 5% and a daily correlation coefficient above 0.99 for all bands. Therefore, the absolute calibration coefficient values were 0.02486 for the Blue band, 0.03554 for the Green band, 0.03575 for the Red band, and 0.02056 for the NIR band.

In order to consolidate the absolute radiometric calibration, parallel cross calibration was performed at an EOS-recommended site in Libya-4, whereby simulated 6S and SBAF compensated Landsat 8 reflectance was used to determine the calibration coefficients for KOMPSAT-3A. The percentage difference was within 4% in the visible band and above 5% in the NIR band, attributed to the 940 nm water absorption band-dependent RSR effect of KOMPSAT-3A. Additionally, analyzing the calibration Gains and TOA radiance revealed that the results of this study displayed less than 5% difference in all bands compared to prelaunch, while the cross calibration Gain was below 5% in the visible band and above 5% in the near infrared band.

Two absolute calibration image scanning modes were applied in this study. We applied absolute scanning mode 1 for vicarious calibration and mode 2 for cross calibration. The blue band was stable for both modes, while the other bands’ coefficients almost doubled in mode 1. 

However, we analyzed in detail the various uncertainties in this approach emanating from tarp reflectance, satellite DN, atmospheric conditions (vapor and visibility), BRDF, and ozone parameters. Therefore, the overall uncertainty was 4.27%, gradually ascending from Blue, Green, and NIR, to Red. However, the Korean site registered a value of 5.39%, as opposed to the Mongolian site, which had 3.73% total uncertainties.

It should be noted that absolute radiometric calibration incorporating vicarious and cross-calibration methods significantly enhances the sensor monitoring and data quality. Therefore, this study provides fundamental data for maintaining the radiometric integrity of KOMPSAT-3A in the absence of an onboard calibrator. However, subsequent AOD and ozone levels must be meticulously quantified using a sky radiometer and sun photometer. The derived radiometric calibration coefficients for KOMPSAT-3A will be officially published by KARI.

## Figures and Tables

**Table 1:** Image list and metadata used.

Site (Latitude, Longitude)	Date	Overpass Time (UTC)	Sun		Satellite			
			Zenith Angle (deg)	Azimuth Angle (deg)	Altitude (km)	Tilt Angle (deg)	Zenith Angle (deg)	Azimuth Angle (deg)
Goheung (34.6 N, 127.2 E)	26 May 2015	04:45:10	23.2	240.2	527.4	30.2	33.0	262.8
	27 May 2015	04:53:50	21.3	236.3	527.1	9.2	9.9	261.4
	28 May 2015	04:43:42	19.4	231.9	526.8	14.9	16.2	80.0
Zuunmod (47.7 N, 107.0 E)	17 June 2015	05:57:17	27.5	231.5	532.0	17.3	18.8	260.5
	18 June 2015	05:47:01	26.6	208.7	532.5	2.5	2.8	78.6
	19 June 2015	05:36:46	25.8	203.5	533.0	21.5	23.4	76.7

**Table 2:** KOMPSAT-3A characteristics and the Second Simulation of Satellite Signal in the Solar Spectrum (6S).

Parameter	LANDSAT-8 (nm)	KOMPSAT-3A AEISS-A (nm, W·m^−2^·band^−1^)						6S Libya-4 (28.9N, 23.8E)
Band	Nominal	Nominal	50%	Bandwidth	Center	ESUN	KOMPSAT-3A RSR and ESUN	
Blue	452~512	450~520	445.6~532.3	86.7	489.0	2001.28		
Green	533~590	520~600	511.9~606.1	94.2	559.0	1875.48		
Red	636~673	630~690	633.1~702.8	69.7	668.0	1524.25		
NIR	851~879	760~900	756.0~932.2	176.2	844.1	1027.38		
Altitude	705 km	528 km						528 km

**Table 3:** Atmospheric input parameters employed for absolute calibration.

	Goheung (2015)						Zuunmod (2015)		
	May 26		May 27		May 28		Jun 17	Jun 18	Jun 19
Aerosol model	Rural		Rural		Rural		Desert	Desert	Desert
OMI ozone (DU)	312.9		314.1		311.7		336.2	335.6	336.8
Field/AWS	Field	AWS	Field	AWS	Field	AWS	AWS	AWS	AWS
Visibility (km)	18.8	18.8	18.7	20	13.6	20	39	36	26
Water vapor (mb)	13.8	13.8	14.2	8.2	12.3	9.7	5.9	5.9	5
Temperature (°C)	30.9	30.9	29.2	27.8	27.2	26	27.9	25.3	28.2
Pressure (hPa)	1009	1009	1010	1008	1008	1010	840.3	842	843.9
Humidity (%)	31.0	31	35.0	22	34.0	29	15.6	18.3	13.2

**Table 4:** Daily absolute calibration results calculated using linear regression between the digital number (DN) and the top of atmosphere (TOA) radiance (GH: Goheung, ZM: Zuunmod).

Site	Date	Blue		Green		Red		NIR	
		Gain	R2	Gain	R^2^	Gain	R^2^	Gain	R^2^
GH	05/26	0.02447 ± 4.9 × 10^−4^	0.999	0.03430 ± 7.9 × 10^−4^	0.998	0.03406 ± 53.0 × 10^−4^	0.999	0.02045 ± 4.7 × 10^−4^	0.998
	05/27	0.02495 ± 3.8 × 10^−4^	0.999	0.03594 ± 5.5 × 10^−4^	0.999	0.03648 ± 51.0 × 10^−4^	0.999	0.02004 ± 5.5 × 10^−4^	0.998
	05/28	0.02529 ± 3.7 × 10^−4^	0.999	0.03605 ± 4.8 × 10^−4^	0.999	0.03558 ± 32.0 × 10^−4^	0.999	0.02090 ± 4.1 × 10^−4^	0.999
ZM	06/17	0.02538 ± 7.9 × 10^−4^	0.999	0.03639 ± 8.5 × 10^−4^	0.999	0.03703 ± 18.7 × 10^−4^	0.998	0.02068 ± 6.3 × 10^−4^	0.999
	06/18	0.02479 ± 5.3 × 10^−4^	0.999	0.03560 ± 7.9 × 10^−4^	0.999	0.03662 ± 16.4 × 10^−4^	0.998	0.02067 ± 7.1 × 10^−4^	0.999
	06/19	0.02407 ± 1.7 × 10^−4^	0.999	0.03529 ± 6.0 × 10^−4^	0.999	0.03548 ± 9.2 × 10^−4^	0.999	0.02076 ± 8.7 × 10^−4^	0.998

**Table 5:** The absolute calibration coefficients at Goheung and Zuunmod.

Band	All	R^2^	Goheung	Zuunmod	Standard Deviation	Difference (%)^(1)^
Blue	0.02486 ± 0.00020	0.9992	0.02489 ± 0.00024	0.02479 ± 0.00037	0.004	0.40
Green	0.03554 ± 0.00031	0.9990	0.03532 ± 0.00042	0.03580 ± 0.00041	0.007	−1.35
Red	0.03575 ± 0.00039	0.9984	0.03536 ± 0.00043	0.03638 ± 0.00057	0.011	−2.88
NIR	0.02056 ± 0.00021	0.9987	0.02046 ± 0.00028	0.02070 ± 0.00033	0.003	−1.17

(1) (Goheung − Zuunmod)/Goheung × 100.

**Table 6:** Comparison of TOA radiance accuracy assessment for simulated 6S and observed K3A TOA radiance using the initial absolute calibration coefficient [10].

Band		Blue	Green	Red	NIR
Observed Radiance (W·sr^−1^·m^−2^)	KOMPSAT-3A (mean ± stdev)	133.93 ± 15.55	162.45 ± 20.03	191.91 ± 24.12	140.93 ± 17.39
Simulated Radiance (W·sr^−1^·m^−2^)	6S (mean ± stdev)	138.08 ± 15.14	170.01 ± 18.65	198.33 ± 21.75	129.16 ± 14.17
Correlation	Similarity^(1)^ (%)	103.22	104.89	103.93	91.91
	R2	0.9168	0.9427	0.9572	0.9535
Radiance Difference ^(2)^ (W·sr^−1^·m^−2^)	Average	−4.15	−7.56	−6.42	11.77
	Stdev	4.65	4.51	4.98	4.38

(1) 6S/KOMPSAT-3A * 100, (2) KOMPSAT-3A − 6S.

**Table 7:** Comparison of TOA reflectance for each band of Landsat-8 (L8) and KOMPSAT-3A (K3A) by spectral band adjustment factor (SBAF) (unit: %).

Band	L8 (a)	Without SBAF K3A (b)	Without SBAF Reflectance Difference ^(1)^	K3A SBAF Coefficient	With SBAF K3A (c)	With SBAF Reflectance Difference^(2)^
Blue	0.251	0.248	−0.003 (−1.19%)	0.979	0.243	−0.008 (−3.27%)
Green	0.335	0.323	−0.012 (−3.65%)	1.014	0.328	−0.007 (−2.23%)
Red	0.466	0.432	−0.034 (−7.78%)	1.023	0.442	−0.024 (−5.16%)
NIR	0.596	0.488	−0.108 (−22.04%)	1.221	0.596	−0.000 (−0.03%)

(1) (b) − (a), (2) (c) − (a).

**Table 8:** The cross calibration vs. absolute coefficients.

Band	Cross Calibration		Absolute vs Cross Calibration Difference		
	Mode1	Mode2	Average		Stdev.
Blue	0.02566 ± 0.00089	0.02566 ± 0.00089	−0.00080	−3.12%	0.00089
Green	0.03727 ± 0.00110	0.01863 ± 0.00055	−0.00086	−4.61%	0.00055
Red	0.03703 ± 0.00104	0.01852 ± 0.00052	−0.00064	−3.46%	0.00052
NIR	0.01888 ± 0.00050	0.00944 ± 0.00025	0.00084	8.90%	0.00025

**Table 9:** Comparison of the calibration Gain coefficients with other research.

Band	Blue	Green	Red	NIR
Prelaunch^(1)^	0.02571	0.03755	0.0358	0.02115
Absolute/Prelaunch	0.967	0.946	0.998	0.972
Absolute/Cross calibration^(2)^	0.969	0.954	0.965	1.089

(1) [10], (2) Mode 2 cross calibration.

**Table 10:** Uncertainty of tarp DN in satellite images and the bidirectional reflectance distribution function (BRDF) (unit: %).

	DN					BRDF				
Date	Blue	Green	Red	NIR	Mean	Blue	Green	NIR	Red	Mean
26 May 2015	1.90	2.54	2.68	3.83	2.50	0.89	1.02	0.14	0.40	0.85
27 May 2015	2.99	1.98	2.60	2.78	2.34	0.25	0.38	0.71	0.58	0.56
28 May 2015	1.92	3.61	2.97	3.24	2.52	1.63	1.49	0.97	1.27	1.23
17 June 2015	0.81	0.96	1.16	1.53	1.23	0.41	0.42	0.39	0.50	0.44
18 June 2015	0.83	1.06	1.47	1.79	1.43	0.51	0.45	0.45	0.48	0.47
19 June 2015	1.02	0.98	1.07	1.27	1.18	0.44	0.47	0.41	0.42	0.44
Mean	1.81	2.14	2.24	2.70	2.06	0.68	0.70	0.50	0.60	0.66

**Table 11:** TOA radiance uncertainty due to atmospheric simulations (unit: %).

	3.5% Reflectance Tarp	23% Reflectance Tarp	32% Reflectance Tarp	53% Reflectance Tarp	Mean
Blue	0.560	0.690	0.824	0.749	0.699
Green	0.657	0.668	0.773	0.629	0.687
Red	0.379	0.599	0.684	0.621	0.587
NIR	0.342	0.435	0.539	0.504	0.468
Mean	0.518	0.598	0.697	0.635	0.614

**Table 12:** Total uncertainty (unit: %).

	5/26	5/27	5/28	6/17	6/18	6/19	ALL
Blue	4.01	4.71	5.12	3.76	3.66	3.71	4.11
Green	4.39	4.17	6.04	3.80	3.71	3.70	4.27
Red	5.17	4.63	5.27	3.99	3.98	3.79	4.44
NIR	4.36	4.63	5.33	3.85	3.84	3.72	4.30
ALL	4.43	4.51	5.39	3.84	3.78	3.73	4.27
